# Inhibition of ovarian cancer cell proliferation by Pien Tze Huang via the AKT-mTOR pathway

**DOI:** 10.3892/ol.2014.1989

**Published:** 2014-03-21

**Authors:** FAN HE, HUI-NI WU, MU-YAN CAI, CHANG-PENG LI, XIN ZHANG, QUAN WAN, SHUANG-BO TANG, JIAN-DING CHENG

**Affiliations:** 1 Department of Forensic Medicine, Zhongshan Medical School, Sun Yat-Sen University, Guangzhou, Guangdong 510080, P.R. China; 2 Department of Preventive Medicine, School of Public Health, Sun Yat-Sen University, Guangzhou, Guangdong 510080, P.R. China; 3 Department of Cancer Research, State Key Laboratory of Oncology in South China, Cancer Center, Sun Yat-Sen University, Guangzhou, Guangdong 510080, P.R. China; 4 Shanghai University of Traditional Chinese Medicine, Shanghai 201203, P.R. China; 5 Guanghua School of Stomatology, Sun Yat-Sen University, Guangzhou, Guangdong 510080, P.R. China

**Keywords:** Pien Tze Huang, ovarian cancer, proliferation, cell cycle

## Abstract

Pien Tze Huang (PZH) is a well-known Chinese medicine that has been used as a therapeutic drug in the treatment of a number of diseases, such as hepatocellular carcinoma and colon cancer. However, few studies have analyzed the effects of PZH on ovarian cancer cell proliferation. In the present study, 3-(4,5-dimethylthiazol-2-yl)-2,5-diphenyltetrazolium bromide and Transwell assays, cell cycle and apoptosis rate analyses and western blotting were conducted to investigate the effects of PZH on the proliferation rate of ovarian cancer cells and its potential molecular pathway. The results showed that PZH inhibits the proliferation of the human ovarian cancer OVCAR-3 cell line by blocking the progression of the cell cycle from the G_1_ to S phase, however, PZH did not induce OVCAR-3 cell apoptosis. Increased PZH concentration may downregulate the expression of AKT, phosphorylated (p)-AKT, mammalian target of rapamycin (mTOR) and p-mTOR proteins in the OVCAR-3 cell line. In addition, it was observed that PZH may suppress the protein expression of cyclin-dependent kinase (CDK)4 and CDK6. Overall, the results of the present study indicated that PZH may inhibit ovarian cancer cell proliferation by modulating the activity of the AKT-mTOR pathway.

## Introduction

Ovarian cancer is the fifth leading cause of cancer-related mortality in females and is a fatal disease among all gynecologic malignancies. The main difficulty in curing ovarian cancer is that the majority of patients are diagnosed at an advanced stage. According to previous studies, as many as 70% of patients have already reached stages III or IV of the disease at the time of diagnosis (1). Due to the latent onset of ovarian cancer, no effective medical screening methods have yet been identified for the improved identification and detection of the disease. In addition, the current clinical therapy for ovarian cancer is optimal primary cytoreductive surgery, followed by systemic chemotherapy with a combination of paclitaxol and platinum (2). However, due to the side effects of these medicines and the increasing tolerance of ovarian cancer to chemotherapy, the efficacy of chemotherapy is limited. Traditional Chinese medicine (TCM) has been used in China for >1,000 years as an anticancer treatment for a number of different malignancies, including liver, lung and hematopoietic cancers (3–5). TCM is considered to induce few side-effects and little tumor cell resistance and has recently been recognized as a key source of novel drugs for molecular therapies. Clinical practices have also shown that a number of TCMs exhibit antitumor activity, which provides a novel therapeutic strategy for cancer treatment (6). Pien Tze Huang (PZH) is a well-known TCM that was identified ~450 years ago and has been applied to the treatment of liver diseases, cancer, stroke and inflammation (7). Similar to a number of other TCMs, PZH contains numerous ingredients, including musk, calculus bovis, snake’s gall and tienchi. As for its application as a cancer treatment, PZH has been widely used in China and Southeast Asia for centuries as a folk remedy for various types of cancer, due to its temperature reducing and detoxification effects. Recent studies have also demonstrated that PZH exhibits therapeutic effects in clinical trials of tumors, such as hepatocellular carcinoma and colon cancer. In addition, it has been reported that PZH inhibits the growth of gastric carcinoma, colon cancer and hepatoma *in vivo* and *in vitro* (8–10). However, the complexity of the ingredients and the obscurity of its mechanism of action have inhibited the wider use of PZH. In the current study, a 3-(4,5-dimethylthiazol-2-yl)-2,5-diphenyltetrazolium bromide (MTT) assay was performed to investigate the effects of PZH on the cell viability of the human ovarian cancer OVCAR-3 cell line, and a Transwell assay was conducted to analyze the effects of PZH on cell invasiveness. Hoechst 33258 staining was also performed to detect the apoptosis of the OVCAR-3 cells. In addition, flow cytometry was conducted to examine the apoptosis frequency and cell cycle changes in the OVCAR-3 cells with various PZH concentrations. Finally, western blotting was performed to investigate the effect of PZH on the variation of important signal transduction pathways in the OVCAR-3 cell line. The study was approved by the ethics committee of Sun Yat-Sen University (Guangzhou, China).

## Materials and methods

### Materials and reagents

PZH was purchased from Zhangzhou Pientzehuang Pharmaceutical Co., Ltd., (Zhangzhou, China). PZH solutions were prepared by dissolving PZH powder in double distilled water to a final concentration of 50 mg/ml and then stored at −20°C until use. The working concentrations of PZH were made by diluting the stock solution in the culture medium to concentrations of 250, 500 and 1,000 μg/ml.

### Cell culture

The human ovarian cancer OVCAR-3 cell line was obtained from the Type Culture Collection of the Chinese Academy of Sciences (Shanghai, China). The cells were cultured in RPMI 1640 medium containing 10% (v/v) fetal bovine serum at a temperature of 37°C in a humidified atmosphere of 5% CO_2_. The cells were then subcultured at 80–90% confluence, treated with PZH concentrations of 250, 500 and 1,000 μg/ml for 24 h and harvested for further study.

### MTT assay

The OVCAR-3 cells were seeded into 96-well plates at a density of 5×10^3^ cells/well in 0.1 ml medium. The cells were then treated with consecutive concentrations of PZH (0, 250, 500 and 1,000 μg/ml) for the same time periods. Following 24 h, 100 μl MTT [0.5 mg/ml in phosphate-buffered saline (PBS)] was added to each well and the cells were incubated for an additional 4 h. Next, the MTT formazan precipitate was dissolved in 100 μl dimethyl sulfoxide and the absorbance was measured at 570 nm using an ELISA reader (ST360, Flyde, Guangzhou, China). An optical density (OD) result of zero concentration was used to present 100% cell viability and thus, the cell viability at other concentrations was calculated using the following formula: Cell viability_x_ = OD_x_ / OD_o_, where OD_x_ is the OD of cells treated with a concentration of PZH and OD_o_ is the OD of cells without PZH treatment.

### Transwell assay

For the Transwell assay, Transwell inserts with 8-μm pores were used. The OVCAR-3 cells were harvested and 200 μl cell suspension (1×10^6^ cells/ml) from each treatment was added in triplicate wells. Following 24 h of incubation, the cells that had migrated through the filter into the lower wells were quantified using an MTT assay and expressed as a percentage of the sum of the cells in the upper and lower wells.

### Hoechst 33258 staining

The cells were plated in six-well plates and incubated for 24 h. Concentrations of PZH were then added to each well of the three experimental groups, and then incubated for 24 h together with the negative control group. Next, the cells were washed three times with PBS and stained with Hoechst 33258 (1 mg/l) for 15 min at 37°C. Images of the Hoechst 33258 fluorescence were then captured using inverted fluorescence microscopy (U-CMAD3; Olympus, Tokyo, Japan), prior to washing the cells three times with PBS. The percentage of positively-stained cells was calculated according to the images.

### Wound healing assay

The OVCAR-3 cells were plated in six-well plates and incubated for 24 h. The cells were then scratched with a yellow tip pipette to create a wound and definite scratches in the center of the dishes with a clear field. Next, concentrations of PZH were added to the three experimental groups, and then medium without serum was added to these plates and that of the negative control group. Images of the cells that had migrated from the leading edge were captured following intervals of 0, 4, 8 and 12 h.

### Apoptosis rate and flow cytometry analysis

The OVCAR-3 cells were harvested following exposure to various concentrations of PZH for 24 h. The cells were then centrifuged (500 × g for 5 min), collected and washed with PBS, followed by resuspension in binding buffer. Next, the cells were incubated with 5 μl Annexin V in the dark for 10 min, washed with binding buffer and resuspended in l% formaldehyde at 40°C for 30 min. The cells were then washed again and stained with 500 μl propidium iodide (PI) for 15 min. Finally, the apoptosis rate was determined by flow cytometry and analyzed by BD CellQuest™ software (BD Biosciences, Franklin Lakes, NJ, USA).

### Cell cycle analysis

The cell cycle analysis was performed using flow cytometry (Beckman Coulter, Miami, FL, USA) and PI staining. The ovarian cancer cells treated with 0, 250, 500 and 1,000 μg/ml of PZH were dissociated from the culture plates using a solution of 0.1% trypsin in PBS for 3 min. Next, the OVCAR-3 cells were collected, adjusted to a concentration of 1×10^6^ cells/ml and fixed in 70% ethanol at 4°C overnight. The fixed cells were then washed twice with cold PBS and incubated for 30 min with RNase (8 μg/ml), 0.1% Triton X-100 and PI (10 μg/ml). The fluorescence signal was detected by flow cytometry (Beckman Coulter) through fluorescence channel 2, and the proportion of DNA in each phase was analyzed by Modfit LT version 3.0 (Verity Software House Inc., Topsham, ME, USA).

### Western blotting

The OVCAR-3 cells were treated with various concentrations of PZH for 24 h and then lysed with mammalian cell lysis buffer (RIPA; Thermo Fisher Scientific, Waltham, MA, USA) containing protease and phosphatase inhibitor cocktails (1:1,000) for 30 min on ice. Next, the raw homogenate was centrifuged (13,000 × g) at 4°C for 20 min, and the supernatants (20 μg) with 4× loading buffer were heated for 10 min at 100°C. The proteins were then separated in 12% SDS-PAGE gels and transferred to polyvinylidene fluoride membranes. Membranes were blocked with 5% skimmed milk in 1× Tris-buffered saline with 0.1% Tween-20 for 1 h at room temperature and then incubated overnight with monoclonal antibodies against p53, AKT, AKT-1, phosphorylated (p)-AKT, mammalian target of rapamycin (mTOR), p-mTOR, cyclin-dependent kinase (CDK)4, CDK6 and GAPDH (1:1,000) at 4°C. The appropriate horseradish peroxidase-conjugated secondary antibody was added, followed by enhanced chemiluminescence detection.

### Statistical analysis

All data were analyzed using the SPSS package for windows (version 13.0; SPSS, Inc., Chicago, IL, USA) and statistical analysis of the data was performed using Student’s t-test and a one-way analysis of variance. P<0.05 was considered to indicate a statistically significant difference.

## Results

### PZH inhibits the proliferation of OVCAR-3 cells

**Figure 1:**
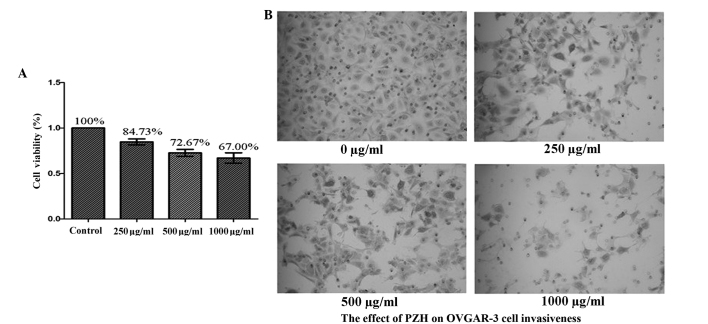
(A) Effect of PZH on the cell viability of OVCAR-3 cells treated with different concentrations of PZH for 24 h. Cell viability was detected by MTT assay and the data were normalized to the viability of the control cells. Data are presented as the mean ± standard deviation from at least three repeated experiments. (B) The effect of PZH on OVCAR-3 cell invasiveness was investigated and the invasive rate of the OVCAR-3 cells was found to decrease with increased PZH concentration. PZH, Pien Tze Huang; MTT, 3-(4,5-dimethylthiazol-2-yl)-2,5-diphenyltetrazolium bromide.

The cell viability of the OVCAR-3 cells was detected by MTT assay to compare the PZH-treated cells with the untreated controls. As shown in Fig. 1, treatment with 250–1,000 μg/ml PZH for 24 h was found to reduce cell viability from 100 to 84.73, 72.67 and 67.00% compared with the control (P=0.002; Fig. 1A).

### PZH inhibits the invasion of OVCAR-3 cells

Treatment with PZH alone marginally decreased the invasion ability of the OVCAR-3 cells. Moreover, the invasion ability of the OVCAR-3 cells was found to markedly decrease with increasing concentrations of PZH (Fig. 1B).

### PZH does not effect the apoptosis of OVCAR-3 cells

**Figure 2:**
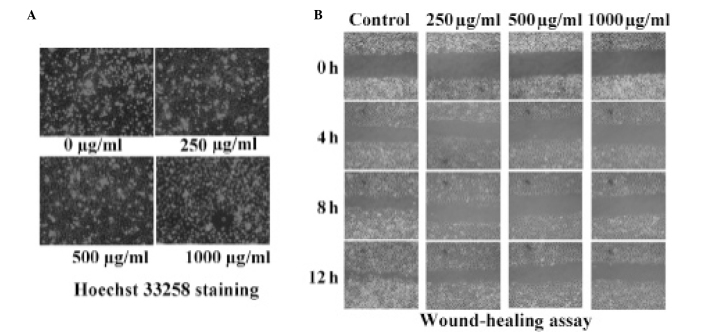
PZH does not induce the apoptosis of OVCAR-3 cells. Following the treatment of the OVCAR-3 cells with the indicated concentrations of PZH for 24 h, (A) PZH was not found to induce apoptosis in the cells and (B) the migration ability of the cells was inhibited with increasing concentrations of PZH, as observed using phase-contrast microscopy (magnification, ×100). Images are representative of three independent experiments. PZH, Pien Tze Huang.

PZH was not found to induce the apoptosis of the OVCAR-3 cells. The percentage of apoptotic cells was calculated using Hoechst 33258 staining, which revealed that the apoptotic rate of the OVCAR-3 cell line did not significantly increase with increasing concentrations of PZH (Fig. 2A).

### PZH-induced inhibition of OVCAR-3 cell migration

A wound-healing assay was performed to investigate the migratory ability of cells treated with various concentrations (0, 250, 500 and 1,000 μg/ml) of PZH. It was demonstrated that the OVCAR-3 cells treated with PZH healed the scratch wounds more rapidly than the negative control group in a dose-dependent manner. As shown in Fig. 2B, increasing concentrations of PZH reduced the migration and invasion ability of the OVCAR-3 cells.

### Effects of PZH on the apoptosis of OVCAR-3 cells

**Figure 3:**
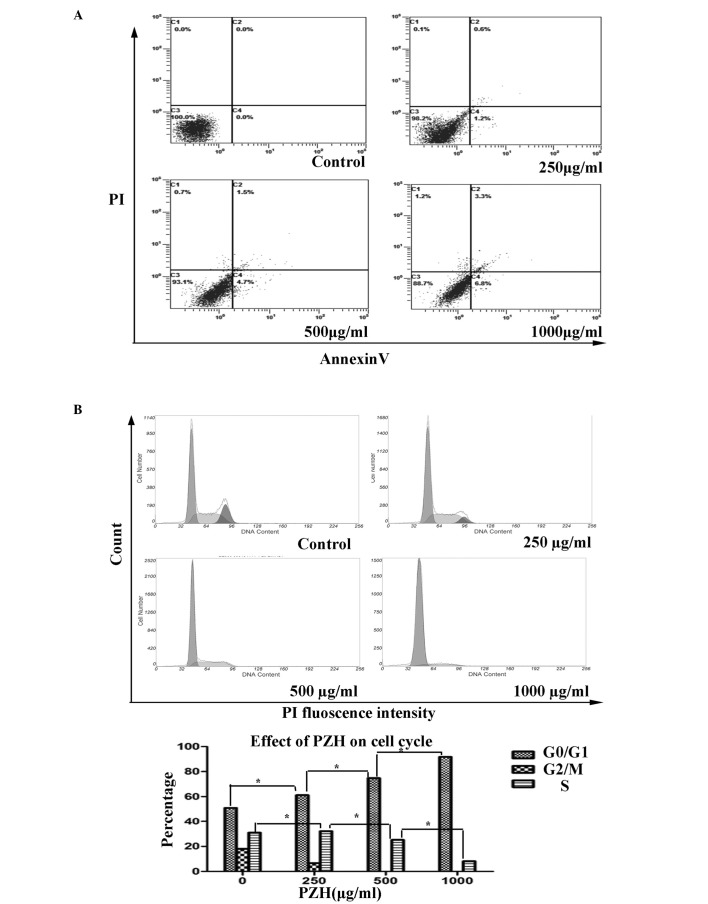
FACs analysis of the apoptosis and cell cycle progression of OVCAR-3 cells. (A) PZH was not found to induce apoptosis in the OVCAR-3 cells collected and stained with Annexin V/PI. Annexin V-positive/PI-negative stained cells (lower right) and Annexin V/PI double-positive stained cells (upper right) represent early and late apoptosis, while Annexin V-negative and PI-positive stained cells (upper left) represent dead cells. Data are presented as the mean ± standard deviation from three independent experiments ^*^P<0.05 vs. control cells. (B) PZH blocked G_1_/S progression in the OVCAR-3 cells that were collected and stained with Annexin V/PI and analyzed by FACS. Following treatment with consecutive concentrations of PZH (0, 250, 500 and 1,000 μg/ml), the number of cells in the G_1_ phase increased by 50.99, 61.23, 74.76 and 91.81%, respectively (P=0.004), while the percentage of cells in S-phase was 30.98, 32.11, 25.243 and 8.19%, respectively (P=0.022). PZH, Pien Tze Huang; FACS, fluorescence-activated cell sorting; PI, propidium iodide.

The percentage of the OVCAR-3 cells undergoing apoptosis was detected by PI staining and determined by fluorescence-activated sorting (FACS) analysis. With the consecutive treatment of the OVCAR-3 cells with concentrations of PZH (0, 250, 500 and 1,000 μg/ml), the apoptosis rates were observed to be 0, 6.6, 30.9 and 43.2%, respectively (P=0.141; Fig. 3A).

### PZH regulates the cell cycle in OVCAR-3 cells

PZH was observed to block G_1_/S phase progression in the OVCAR-3 cells, as determined by PI staining and FACS analysis. With the consecutive treatment of the OVCAR-3 cells with the various concentrations of PZH (0, 250, 500 and 1,000 μg/ml), the number of cells in the G_1_ phase was found to increase, with percentages of 50.99, 61.23, 74.76 and 91.81%, respectively (P=0.004). By contrast, the percentage of cells in the S phase were found to be 30.98, 32.11, 25.24 and 8.19%, respectively (P=0.022; Fig. 3B). These results indicated that PZH may inhibit OVCAR-3 cell proliferation by blocking cell cycle progression from the G_1_ to S phases.

### PZH regulates protein expression in OVCAR-3 cells

**Figure 4:**
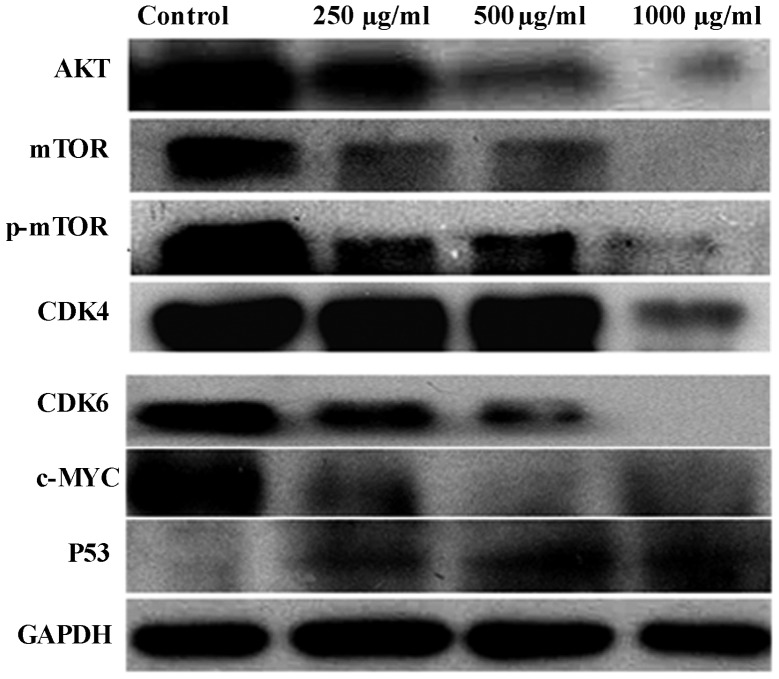
Effect of PZH on the expression of AKT, p-AKT, AKT-1, mTOR, p-mTOR, CDK4, CDK6, p53 and c-Myc in OVCAR-3 cells. Cells were treated with the indicated concentrations of PZH for 24 h and analyzed by western blotting. GAPDH was used as the internal control. Data are representative of three independent experiments. The expression levels of AKT, p-AKT, AKT-1, mTOR, p-mTOR, CDK4, CDK6 and c-Myc were found to decrease compared with the control group. Furthermore, a marked increase in the protein expression of p53 was observed. PZH, Pien Tze Huang; mTOR, mammalian target of rapamycin; CDK, cyclin-dependent kinase; p−, phosphorylated.

PZH regulates the expression of AKT, p-AKT, mTOR, p-mTOR, CDK4, CDK6, p53 and c-Myc in OVCAR-3 cells, and following PZH treatment for 24 h in the present study, the expression levels of these proteins were found to decrease compared with those in the control group. In addition, a marked increase in p53 protein expression was observed (Fig. 4).

## Discussion

The main disadvantage of current cancer chemotherapy is drug resistance in tumor cells and toxicity against normal cells, which largely limits the efficacy of chemotherapy treatments (11). However, it is well acknowledged that TCM, which consists of several herbs, is characterized by few side-effects and limited drug tolerance, thus indicating the potential use of TCM in cancer treatment (12). Previous studies and clinical practice have shown that PZH exhibits an anticancer capacity and has the ability to induce cell apoptosis. However, its effects on cell proliferation and the cell cycle and its underlying molecular mechanism remain unclear. Therefore, the full elucidation of the effect and molecular mechanisms of PZH with regard to its specific anticancer function is required.

The aim of the present study was to investigate the underlying pathway and effect of PZH on the proliferation and cell cycle of OVCAR-3 cells. The FACS analysis revealed that the percentage of OVCAR-3 cells in the S and G_2_ phases of the cell cycle decreased significantly with increasing concentrations of PZH, which indicated that a high concentration of PZH may exhibit a significant anticancer effect via regulation of the cell cycle. In addition, PZH was found to inhibit the migration and invasion ability of the OVCAR-3 cells.

It is well known that cellular growth, proliferation and survival are regulated by a complex network of intracellular and extracellular signal transduction cascades, and that the growth factor-responsive receptor tyrosine kinase (RTK)-phosphatidylinositol 3-kinase (PI3K) pathway is an important mediator of these processes (13). The serine/threonine kinase AKT functions as a central integrator of the RTK-PI3K signaling cascade, which modulates downstream effectors and most notably the tuberous sclerosis complex 1/2-mTOR pathway. Furthermore, mTOR is a serine/threonine protein kinase, termed the ‘target of rapamycin’, which serves as a primary regulator of protein synthesis and cell growth (14). Genetic studies in Drosophila and mice (15–18) have also shown that mTOR activity affects cell size, which is a key parameter that governs entry into the cell cycle (19). mTOR also integrates diverse upstream signals, including amino acid- and energy stress-sensing, to regulate cell proliferation, growth and survival (20,21). Notably, it has also been confirmed that the aberrant stimulation of the PI3K-AKT-mTOR pathway is associated with a poor prognosis in ovarian cancer patients (22). In terms of cell survival rate and cell cycle changes, to improve our understanding of the potential molecular mechanism of PZH, the current study detected the expression levels of total AKT, mTOR and p-mTOR proteins following treatment with various concentrations of PZH. The results revealed that the expression levels of the AKT, mTOR and p-mTOR proteins evidently decreased with increasing concentrations of PZH, which indicated that the AKT-mTOR pathway may be inhibited by PZH.

In addition, the expression of certain downstream proteins of the AKT-mTOR pathway was investigated, and the expression of p53, termed as the ‘guardian of the genome’ (23,24), was evidently upregulated with the increasing concentrations of PZH. It is well known that the p53 protein functions as a cancer suppressor by inhibiting cell growth through cell cycle arrest at the G_1_/S regulation point and initiating apoptosis if the cell is damaged (25). The present results revealed that the expression of specific proteins associated with G_1_/S transition, including cyclin D3, CDK4 and CDK6 (26–28), decreased with the dose-dependent repression of the G_1_/S transition by PHZ, as determined by flow cytometry. Based on the aforementioned PZH-induced apoptosis and reduced cell viability, we propose that inhibition of the PZH-induced OVCAR-3 cell proliferation and G_1_/S transition may be involved in the AKT-mTOR pathway.

In conclusion, the present study indicates that PZH may inhibit the cell proliferation, migration and G_1_/S transition of OVCAR-3 cells via the AKT-mTOR pathway.
